# Maintenance or Collapse: Responses of Extraplastidic Membrane Lipid Composition to Desiccation in the Resurrection Plant *Paraisometrum mileense*

**DOI:** 10.1371/journal.pone.0103430

**Published:** 2014-07-28

**Authors:** Aihua Li, Dandan Wang, Buzhu Yu, Xiaomei Yu, Weiqi Li

**Affiliations:** 1 Key Laboratory of Biodiversity and Biogeography, Kunming Institute of Botany, Chinese Academy of Science, Kunming, China; 2 Plant Germplasm and Genomics Center, Germplasm Bank of Wild Species, Kunming Institute of Botany, Chinese Academy of Sciences, Kunming, China; 3 University of Chinese Academy of Sciences, Beijing, China; Institute of Genetics and Developmental Biology, Chinese Academy of Sciences, China

## Abstract

Resurrection plants usually grow in specific or extreme habitats and have the capacity to survive almost complete water loss. We characterized the physiological and biochemical responses of *Paraisometrum mileense* to extreme desiccation and found that it is a resurrection plant. We profiled the changes in lipid molecular species during dehydration and rehydration in *P. mileense*, and compared these with corresponding changes in the desiccation-sensitive plant *Arabidopsis thaliana*. One day of desiccation was lethal for *A. thaliana* but not for *P. mileense*. After desiccation and subsequent rewatering, *A. thaliana* showed dramatic lipid degradation accompanied by large increases in levels of phosphatidic acid (PA) and diacylglycerol (DAG). In contrast, desiccation and rewatering of *P. mileense* significantly decreased the level of monogalactosyldiacylglycerol and increased the unsaturation of membrane lipids, without changing the level of extraplastidic lipids. Lethal desiccation in *P. mileense* caused massive lipid degradation, whereas the PA content remained at a low level similar to that of fresh leaves. Neither damage nor repair processes, nor increases in PA, occurred during non-lethal desiccation in *P. mileense*. The activity of phospholipase D, the main source of PA, was much lower in *P. mileense* than in *A. thaliana* under control conditions, or after either dehydration or rehydration. It was demonstrated that low rates of phospholipase D-mediated PA formation in *P. mileense* might limit its ability to degrade lipids to PA, thereby maintaining membrane integrity following desiccation.

## Introduction

Drought is a major factor that limits plant growth and yield. In most parts of the world, drought continuously affects crop production and is of growing concern given the increasing demand for food production by the expanding global population [1], [2]. Most crops are sensitive to drought, and except their seeds and pollen grains, their tissues cannot withstand water stress below 20% relative water content (RWC) [3]. However, a small group of so-called resurrection plants can tolerate extreme loss of water (desiccation) to 10% RWC or less [3]. Upon rewatering, the vegetative tissues of resurrection plants can quickly revive from the quiescent state that they enter upon loss of almost all of their free water [4]. Resurrection plants are excellent models to explore the physiological, biochemical, and molecular basis of desiccation tolerance [5]. A better understanding of the unique features of resurrection plants might benefit efforts to improve crop yields under conditions of water deficit.

Resurrection plants exhibit a series of distinct morphological, physiological, biochemical, and genetic protective mechanisms to resist or respond to extreme desiccation. The folding and re-expansion of leaves are the most obvious morphological changes that occur during desiccation and subsequent rewatering; folding might prevent the production of reactive oxygen species (ROS) induced by light during drying and rehydration [4], [6]–[9]. Inward shrinking of the cell wall and dehydration-induced membrane shrinking are typical responses of resurrection plants to desiccation [5], [6], [10]. In these plants, photosynthetic activity is retained during mild drought, is lost during severe desiccation, and returns upon subsequent rehydration [7], [11]–[14]. Resurrection plants do not necessarily share the same physiological strategies, and sometimes even employ completely opposite strategies to deal with extreme desiccation. For example, the osmoprotectant proline is widely used to resist cellular dehydration in plants. However, whereas some resurrection plants accumulate proline following desiccation, others do not [15]–[17]. In addition, some resurrection plants (poikilochlorophyllous species) lose their chlorophyll and degrade their thylakoid membranes to prevent the production of photosynthetically generated ROS during dehydration [9], [18]. Other resurrection plants (homoiochlorophyllous species), such as *Craterostigma plantagineum* and *Haberlea rhodopensis*, retain their chlorophyll and thylakoid structures [19], [20].

The ability of resurrection plants to maintain antioxidant activity even after severe cellular dehydration is thought to account in large part for their distinctive capacity to resist desiccation [9], [21]. Osmoregulatory substances, such as sucrose, alleviate cellular dehydration and oxidative stress in resurrection plants [22]–[25]. Many genes that function in drought tolerance have been cloned from resurrection plants and characterized [26]–[29]. Transformation of certain plants with some of these genes improves drought resistance significantly [30]. The powerful approaches of transcriptomics [31], [32], proteomics [33]–[36], and metabolomics [1] have enabled extensive investigation of the mechanisms that resurrection plants use to resist severe dehydration at the levels of global changes in gene expression and the abundances of proteins and metabolites. Lipid metabolism during and following desiccation was recently reported in *Craterostigma plantagineum* [37]. However, especially given that the tolerance strategies used by resurrection plants are often species-specific, little is known about how molecular species of membrane lipids respond to severe dehydration and subsequent rehydration, and how the changes in lipid profiles contribute to ability to survive extreme desiccation.

Maintenance of membrane integrity and fluidity is of critical importance to ensure that resurrection plants can survive cellular dehydration [5], [38]. Several membrane components, such as phosphatidic acid (PA), sphingolipids, and sterols, have particular effects on membrane permeability [39]–[41]. A widely accepted speculation about the desiccation tolerance of resurrection plants is that although they experience membrane damage during dehydration, they can then repair this damage during their subsequent rehydration [42]. This suggests significant changes in their membrane lipids during both desiccation and rewatering. Three types of lipid change are often observed in resurrection plants during desiccation: 1) decreases in the plastidic lipid monogalactosyldiacylglycerol (MGDG), which is thought to suppress formation of the non-lamellar membrane phase [37], [42]–[44]; 2) increases in fatty acid desaturation, which enhances membrane fluidity and thus favors dehydration resistance [44]–[46]; and 3) increases in PA, which is proposed to be an early signal of cellular dehydration and a structural feature of membrane injury [37], [42], [47]. Many metabolic enzymes regulate the lipid changes induced by cellular dehydration. Phospholipases hydrolyze phospholipids at different positions to produce lyso-phospholipids, diacylglycerol (DAG), or PA [48]. Phospholipases might be the most important enzymes in resurrection plants because they contribute to many aspects of dehydration-induced changes in membrane lipids. For example, the increases in the levels of PA and DAG that occur as levels of other phospholipids decrease during dehydration in *Craterostigma plantagineum* [37] suggest that phopholipases C and D act in response to dehydration. Dramatic increases in the abundances of lyso-phospholipids caused by freezing-induced cellular dehydration [49] suggest roles for phospholipase A and/or B in responses to dehydration. In particular, the role of phospholipase D-mediated PA formation has been extensively studied in processes related to cellular dehydration [49], [50]. However, recent reports indicate that rather than increasing, levels of PA might even decline following desiccation in *A. thaliana* [51]. These findings suggest that there are still unknown responses of lipid changes to dehydration.

*Paraisometrum mileense* W. T. Wang is the only species in the monotypic genus *Paraisometrum* W. T. Wang of family Gesneriaceae. It had been thought to be extinct for one hundred years, but was rediscovered by botanists from the Chinese Academy of Sciences [52]. *P. mileense* is a stemless perennial herb that is 10–20 cm tall [53]. *P. mileense* grows over limestone on rocky outcrops, and in a seasonally arid subtropical region where half of the year is the dry season, and the other half is the wet season; about 88% of the annual rainfall occurs during the wet season, and only about 110 mm of rain falls during the dry season [54]. Generally, resurrection plants can survive extremely harsh environments and are usually found in habitats with sporadic rainfall. These include rocky outcrops and arid zones within tropical and subtropical areas [55]. In general, these plants are small [56], [57]. Among the approximately 300 angiosperm resurrection species, more than two dozen belong to the family Gesneriaceae [58]. Although *P. mileense* is probably highly tolerant of water deficit, it has never been established if it is a resurrection plant.

The present study used physiological and biochemical analyses to demonstrate that *P. mileense* is a resurrection plant. We used lipidomic analyses based on electrospray ionization mass spectrometry (ESI-MS/MS) [49], [59], [60] to explore changes of membrane molecular species in response to dehydration and subsequent rehydration in *P. mileense*, and to compare these with similarly treated desiccation-sensitive *Arabidopsis thaliana* plants. We observed dramatic degradation of membrane lipids during both dehydration and rehydration, but the degradation was markedly different between two treatments in *A. thaliana*. Whereas plastidic lipids were sensitive to non-lethal dehydration in *P. mileense*, extraplastidic lipids were very stable to desiccation. Notwithstanding the dramatic degradation of lipids that occurred upon lethal dehydration of *P. mileense*, the changes differed markedly from those in *A. thaliana*. Whereas both of the major intermediates of lipid metabolism, PA and DAG, were involved in lipid changes in *A. thaliana*, only DAG increased in *P. mileense* during lethal dehydration and rehydration. The responses of fatty acid desaturation and acyl chain length to dehydration and rehydration were also examined in both plant species. We propose that desiccation tolerance might involve avoiding damage and the need for repair, as well as appropriate regulation of phopholipase activities.

## Results

### *P. mileense* tolerates extreme desiccation and is a resurrection plant

To test the desiccation tolerance of *P. mileense*, we reduced the RWC in mature plants by drying them for two, three, four, or five days, and then rehydrating them for 24 h (Figure S1A). The RWC of *P. mileense* seedlings decreased gradually during dehydration. After two days of desiccation, the RWC decreased to about 40%, and then decreased further with additional days of drying, reaching a RWC as low as 3.7%. After rewatering for one day, the seedlings with 3.7% RWC regained their initial RWC, 97.2% (Figure S1C). The leaves curled inward gradually during desiccation and became completely folded until only the abaxial surfaces of the leaves were in the outer whorl; finally, they lost nearly all of their water (Figure S1A, C). As this took place, *F_v_*/*F_m_* (maximal quantum efficiency of photosystem II in the dark adapted state) nearly reached zero (Figure S1A); this indicated that photosynthesis ceased almost completely. During the subsequent day of rehydration, the leaves spread and expanded until the leaves became fully unfolded; eventually, the RWC almost returned to its initial level, at which point the photosynthetic activity returned to the level prior to dehydration. The photosynthetic activity of *P. mileense* leaves recovered to its original level within 24 h after having decreased to a level of nearly zero. For comparison, we also tested the desiccation tolerance of *A. thaliana* under the same conditions. Its leaves quickly lost water and shrank in a random pattern during dehydration. These leaves exhibited a complete loss of chlorophyll fluorescence, which was not recovered during rehydration (Figure S1B). These results show that *P. mileense* could tolerate extreme loss of water below 10% RWC, but that the same conditions were lethal for *A. thaliana*. This demonstrates that *P. mileense* is a resurrection plant.

**Figure 1:**
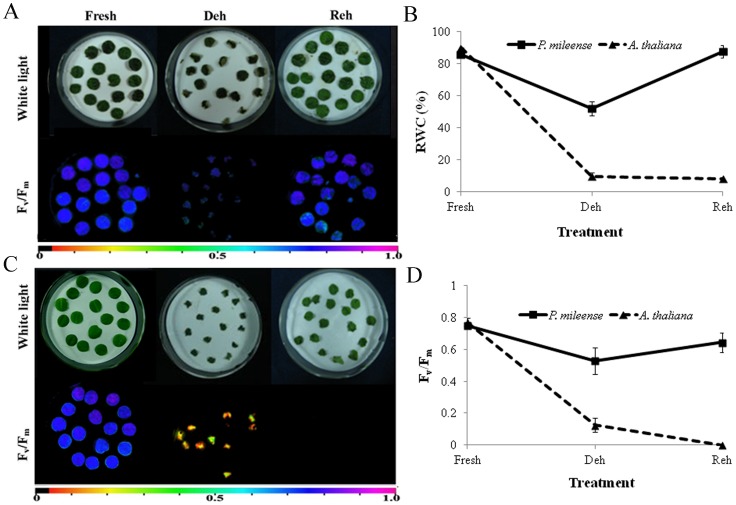
Dehydrated (Deh) and rehydrated (Reh) leaf discs of (A) *P. mileense* and (B) *A. thaliana*. White coloration (upper picture) or low *F_v_*/*F_m_* values for variable fluorescence (lower picture). The color bar at the bottom indicates *F_v_*/*F_m_* values. (C) Relative water content (RWC). (D) *F_v_*/*F_m_*. Values are means ± standard deviation (*n* = 4 or 5).

Generally, leaves of resurrection plants survive desiccation only when they are dried on the plant, although the detached leaves in some resurrection plants also tolerate dehydration [11], [46], [61], [62]. To test whether the detached leaves of *P. mileense* could tolerate dehydration, we dried leaf discs of *P. mileense* and *A. thaliana* side-by-side for one day and then rehydrated them for one day (Figure 1). The RWC of *P. mileense* discs decreased to 52% and then returned to the initial levels; their photosynthetic activity decreased to 0.53 of *F_v_*/*F_m_* value and then recovered to the level associated with normal photosynthetic function. For *A. thaliana* discs, in contrast, RWC decreased to 9.8% and did not recover; their photosynthetic activity was lost completely (Figure 1). We also examined changes in the RWCs of *P. mileense* and *A. thaliana* discs during the processes of complete desiccation and subsequent rehydration (Figure S2); the patterns of change of RWC of leaf discs were the same as those in intact plants (Figure S1C). Given the demonstrated ability of detached *P. mileense* leaves to be resurrected upon rehydration, our subsequent studies focused exclusively on leaf discs of *P. mileense*.

**Figure 2:**
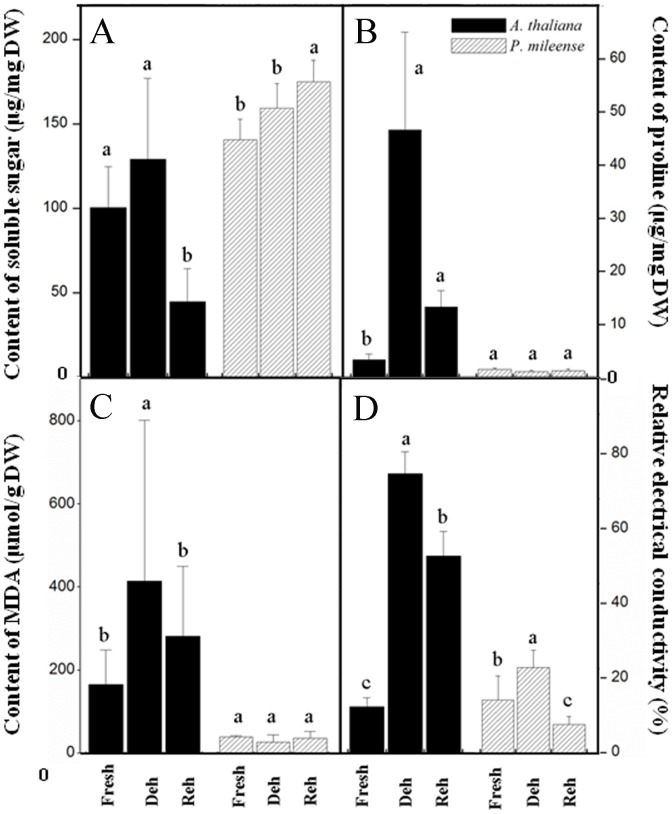
Changes in (A) soluble sugar, (B) proline, (C) malondialdehyde (MDA), and (D) relative electricity conductivity during dehydration (Deh) and rehydration (Reh) in *P. mileense* and *A. thaliana* leaves. Values are means ± standard deviation (*n*  =  4 or 5). Values in the same bar type with different letters indicate that values are significantly different (*P* < 0.05).

We further characterized the resurrection of *P. mileense* by examining the contents of proline, soluble sucrose, and chlorophyll, as well as the changes in membrane lipid peroxidation and ion leakage during dehydration and subsequent rehydration. These were compared with those of similarly treated *A. thaliana* leaf discs. Slight accumulation of sucrose occurred in *P. mileense*, whereas the proline content remained low and unchanged during both dehydration and rehydration (Figure 2A, B). These findings are the same as those for other resurrection plants [11], [22], [24]. The changes in the peroxidation of membrane lipids, which were indicated by the amount of malondialdehyde (MDA) and ion leakage, were significantly lower in *P. mileense* than in *A. thaliana* (Figure 2C, D). These findings indicate that *P. mileense* suffered less damage than *A. thaliana* under the same stress conditions. These lines of evidence affirm the resurrection of *P. mileense* upon rehydration after extreme desiccation. The amounts of chlorophyll (a+b) and carotenoid (c+x) of *P. mileense* leaves decreased by half in comparison with those of the control under dark desiccation, but then returned to the control level after rewatering (Table 1). This means that *P. mileense* is a homoiochlorophyllous resurrection plant.

**Table 1:** Changes of pigments during dehydration (Deh) and rehydration (Reh) in *P. mileense* and *A. thaliana* leaves.

Pigment Class	Species	Pigment content (µg/mg)		
		Fresh	Deh	Reh
**Chl a**	*A. thaliana*	9.26±0.55^b^	7.57±0.42^c^	10.76±1.67^a^
	*P. mileense*	4.98±0.39^a^	2.92±0.99^b^	4.74±0.67^a^
**Chl b**	*A. thaliana*	3.10±0.19^b^	2.49±0.17^c^	4.31±0.62^a^
	*P. mileense*	1.83±0.23^a^	0.88±0.14^b^	1.88±0.27^a^
**Chl a/b**	*A. thaliana*	2.99±0.02^a^	3.04±0.05^a^	2.50±0.04^b^
	*P. mileense*	2.73±0.18^ab^	2.85±0.11^a^	2.54±0.01^b^
**Chl a+b**	*A. thaliana*	12.36±0.74^b^	10.07±0.58^c^	15.07±2.29^a^
	*P. mileense*	6.81±0.60^a^	3.39±0.58^b^	6.62±0.94^a^
**Carotenoid**	*A. thaliana*	1.97±0.10^b^	1.79±0.09^b^	2.32±0.23^a^
	*P. mileense*	1.06±0.10^a^	0.62±0.09^b^	1.13±0.07^a^

Values in the same row with different letters are significantly different (*P*<0.05). Values are means ± SD (*n* = 4 or 5).

### Comparative profiling of molecular species of membrane lipids in leaves of *P. mileense* and *A. thaliana* under the same conditions of dehydration and rehydration

**Figure 3:**
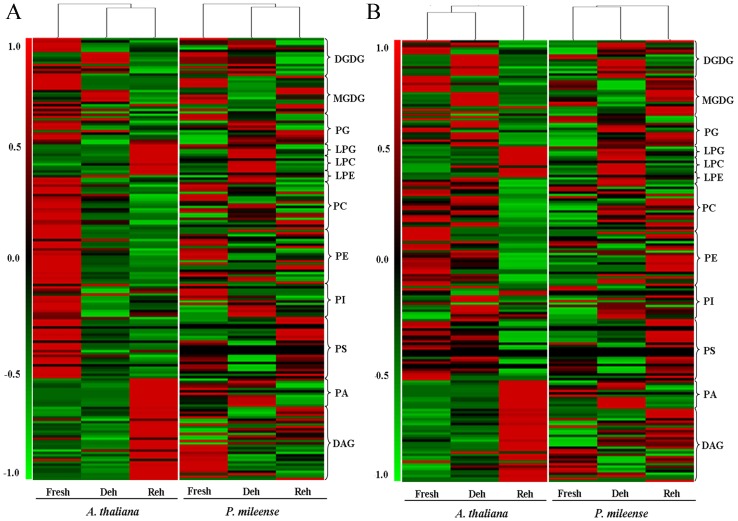
Hierarchical clustering analysis of lipid molecular species during dehydration (Deh) and rehydration (Reh) of *P. mileense* and *A. thaliana* leaves. (A) Contents (nmol/mg dry weight) of lipid molecular species. (B) Compositions (mol %) of lipid molecular species. The colored bar within a column represents the lipid molecular species in the corresponding plants and treatments. The color of each bar represents the abundance of the indicated lipid species, which is expressed relative to the change from the mean center of each lipid species within all treatments. Lipid species in the corresponding lipid classes were sorted using class (as indicated), total acyl carbons (within a class), and total double bonds (with class and total acyl carbons) in ascending order. Values are means (*n* = 4 or 5).

To analyze the changes in membrane lipids during desiccation, we first profiled the lipid molecular species of *P. mileense* and *A. thaliana* under control conditions (fresh leaves) and then after parallel treatment with dehydration for one day and subsequently rehydration for one day (Figure 1). More than 180 molecular species belonging to 12 classes of glycerolipids were quantified. The glycerolipids included six classes of phospholipids: phosphatidylglycerol (PG), phosphatidylcholine (PC), phosphatidylethanolamine (PE), phosphatidylinositol (PI), phosphatidylserine (PS), and PA; two classes of galactolipids: MGDG and digalactosyldiacylglycerol (DGDG); three classes of lyso-phospholipids: LysoPG, LysoPC, and LysoPE; and one class of neural glycerolipid: DAG. Every molecular species was presented as “acyl carbon atoms: double bonds” [63]. The changes in both the contents (nmol/mg dry weight, Figure 3A) and the composition (mol%, Figure 3B) of the lipids were subjected to clustering analysis. Total lipid amounts of each head-group class are shown in Table 2 and Table S1.

**Table 2:** Amount of lipid in each head-group class and total polar lipid during dehydration (Deh) and rehydration (Reh) of *P. mileense* and *A. thaliana* leaves.

Lipid class	Species	Lipid/dry weight (nmol/mg)				
		Fresh	Deh	Reh	RC(F–D)(%)	RC(D–R)(%)
**DGDG**	*A. thaliana*	54.35±2.94^a^	22.19±5.96^b^	4.61±1.15^c^	−59	−79
	*P. mileense*	23.37±0.63^a^	25.37±2.23^a^	19.62±2.42^b^	-	−23
**MGDG**	*A. thaliana*	261.4±15.8^a^	42.05±10.72^b^	5.62±0.56^c^	−84	−87
	*P. mileense*	58.19±2.32^a^	37.3±5.08^b^	37.34±6.98^b^	−36	-
**PG**	*A. thaliana*	23.95±2.44^a^	4.08±1.28^b^	3.02±0.18^b^	−83	-
	*P. mileense*	3.50±0.43^a^	3.90±0.12^a^	3.30±0.27^b^	-	−15
**PC**	*A. thaliana*	26.64±1.91^a^	5.87±1.70^b^	0.36±0.15^c^	−78	−94
	*P. mileense*	6.50±0.71^a^	5.82±1.20^b^	5.42±1.18^b^	−10	-
**PE**	*A. thaliana*	5.75±0.47^a^	0.95±0.32^b^	0.01±0.00^c^	−84	−99
	*P. mileense*	1.15±0.16^a^	0.93±0.15^b^	0.87±0.08^b^	−19	-
**PI**	*A. thaliana*	7.00±0.30^a^	2.93±0.15^b^	1.82±0.31^c^	−58	−38
	*P. mileense*	2.61±0.27^a^	2.62±0.23^a^	2.15±0.37^b^	-	−18
**PS**	*A. thaliana*	0.72±0.15^a^	0.14±0.06^b^	0.00±0.00^c^	−80	−97
	*P. mileense*	0.14±0.04^b^	0.09±0.02^c^	0.21±0.08^a^	−38	142
**PA**	*A. thaliana*	1.17±0.13^b^	0.43±0.10^b^	6.18±2.22^a^	-	1332
	*P. mileense*	0.50±0.05^a^	0.57±0.07^a^	0.31±0.08^b^	-	−45
**LPG**	*A. thaliana*	0.25±0.06^b^	0.33±0.12^b^	4.04±3.42^a^	-	1140
	*P. mileense*	0.05±0.01^a^	0.06±0.02^a^	0.03±0.02^b^	-	−47
**LPC**	*A. thaliana*	0.09±0.01^b^	0.10±0.01^b^	0.27±0.08^a^	-	179
	*P. mileense*	0.04±0.01^b^	0.09±0.03^a^	0.03±0.00^b^	127	−69
**LPE**	*A. thaliana*	0.10±0.01^a^	0.03±0.01^b^	0.03±0.01^b^	−70	-
	*P. mileense*	0.03±0.01^b^	0.06±0.02^a^	0.03±0.01^b^	119	−54
**DAG**	*A. thaliana*	14.71±7.22^b^	26.10±7.47^b^	102.8±26.4^a^	-	294
	*P. mileense*	6.80±1.31^a^	9.75±1.77^a^	8.44±2.70^a^	-	-
**Total polar lipid**	*A. thaliana*	381.4±20.4^a^	78.85±19.43^b^	26.73±6.80^c^	−79	−66
	*P. mileense*	92.80±9.10^a^	77.08±8.33^b^	69.41±10.86^b^	−17	-

The percentage relative change in lipids of dehydration RC (F–D) is the value for the difference between the values of Fresh and Deh discs, divided by the value of Fresh discs; that of rehydration RC (D–R) is the value for the significant difference between the values of Deh and Reh discs, divided by the value of Deh discs. Values in the same row with different letters are significantly different (*P*<0.05). Values are means ± standard deviation (*n* = 4 or 5).

An overview of the lipid contents (Figure 3A) and compositions (Figure 3B) indicated four trends of lipid levels in the response to dehydration and rehydration. 1) There were clear differences between *P. mileense* and *A. thaliana*. These included differences between their fresh leaves and between their leaves subjected to dehydration and rehydration. 2) The changes in *P. mileense* were much smaller than those in *A. thaliana.* 3) Clustering of the lipid contents of dehydrated and rehydrated leaves (Figure 3A) suggested that the treatments caused significant changes in the relative abundances of lipids. 4) The lipid compositions of fresh and dehydrated leaves clustered together (Figure 3B); in other words, both species showed similar changes in lipid compositions after rehydration when these were compared with those of fresh leaves. This indicates the complexity of lipid metabolism in *P. mileense*, and that rewatering might not have simply restored the lipid composition back to that found in fresh leaves prior to dehydration. By contrast, in *A. thaliana*, major membrane damage occurred during rehydration but not during dehydration. The overall abundance of membrane lipids in *P. mileense* was significantly lower than that of *A. thaliana* (Table 2). This is consistent with a previous report that the lipid contents of resurrection plants are usually low [64]. The results of detailed data mining are presented in subsequent sections.

### Differential degradation of membrane lipids occurred during lethal dehydration and subsequent rehydration of *A. thaliana*

After dehydration of *A. thaliana* leaves to 10% RWC, we found that the levels of seven of the twelve classes of lipid studied (including MGDG, PG, PC, PE, PI, PS, and LysoPE), decreased to less than half of their levels prior to drying; the abundances of four classes of lipid (PA, DAG, LysoPG, and LysoPC) remained unchanged; and one class of lipid (DGDG) became more abundant (Table 2). The increase in DGDG levels might have been due to galactosylation of MGDG; this would be consistent with the responses of other species to desiccation [37]. Upon subsequent rehydration, we found that the lipids that degraded during dehydration continued to decrease, and that the rates of degradation increased. Their relative changes during rehydration were greater than those during dehydration (Table 2). In contrast, lipid levels that were maintained during dehydration increased dramatically. Among these, PA and DAG increased 13.3-fold (from 0.44 to 6.18 nmol/mg) and 3.9-fold (from 26.1 to 102.76 nmol/mg), respectively (Table 2).

To further examine which lipids were degraded, we compared acyl structures among the molecular species during rehydration to monitor potential turnover reactions [63]. We found that all degraded molecular species corresponded to the increases of PA molecular species with the same acyl structures, except for the individual PS molecular species that were present at very low levels (Figure S3B). For example, the decrease of 34∶6 MGDG specifically corresponded to the increase of 34∶6 PA; this suggests that 34∶6 MGDG was converted to 34∶6 PA. This is consistent with our previously described observations following freezing-induced dehydration of *A. thaliana* [49]. Interestingly, however, the acyl structures of increased DAG molecular species did not correspond to those of any other decreased lipid molecular species. This suggests that DAG was not directly derived from the major membrane lipids. These results indicate that membrane lipids of *A. thaliana* are degraded dramatically during both desiccation and rewatering, but that their degradation patterns varied and that most degradation occurred during rewatering. The results also suggest that the membrane lipids were degraded directly through PA and indirectly through DAG.

### Extraplastidic membrane lipids essentially remained unchanged during dehydration and rehydration in *P. mileense*

Desiccation of *P. mileense* to 52.1% RWC (Figure 1C) caused only a small (17%) decrease in total membrane lipids (from 97.80 to 77.02 nmol/mg), which was much less than that observed in *A. thaliana* given the same treatment (Table 2). This change was mainly attributable to the decrease in the plastidic lipid MGDG (Table 2), whereas the abundances of other lipids remained essentially unchanged. In terms of the same acyl structures of molecular species, we found that changes in the levels of degraded MGDG corresponded to increases in the levels of DGDG and DAG (Figure S3A). This suggests the possible involvement of the lipid metabolism reaction mediated by SENSITIVE TO FREEZING 2 (SFR2), a galactolipid:galactolipid galactosyltransferase that reduces levels of the plastidic lipid MGDG. The decrease in the level of MGDG also increased the DGDG/MGDG ratio (Table S1), which favored plant survival following the imposition of water deficit [65]. The sensitivity of MGDG levels in *P. mileense* to drought is consistent with observations in other species [37], [42]–[44], [66]. However, the failure of levels of PA and DAG to increase substantially upon dehydration differs from observations made for other resurrection plants [37], [42], [47]. The absence of a change in the PC/PE ratio in *P. mileense* (Table S1) also distinguishes drought adaptation in *P. mileense* from the process in other plants [43], [67], [68]. During rewatering, the levels of both plastidic and extraplastidic lipids were maintained (Table 2), except for certain individual lipids, such as PA and PS. Levels of PA decreased very little, and basically remained at a very low level close to those found in fresh leaves (Table 2). The MGDG level remained low and exhibited no recovery following rehydration. These phenomena differ from those in other resurrection plants after rewatering [37]. Overall, these results indicate that levels of MGDG decreased in *P. mileense* in response to desiccation, but that levels of extraplastidic membrane lipids were maintained throughout the process of dehydration and rehydration; it also suggested that the immediate recovery of MGDG during rewatering was not required to restore viability after desiccation.

### Membrane lipids became more unsaturated and the lengths of acyl chains were maintained during dehydration and subsequent recovery in *P. mileense*

**Figure 4:**
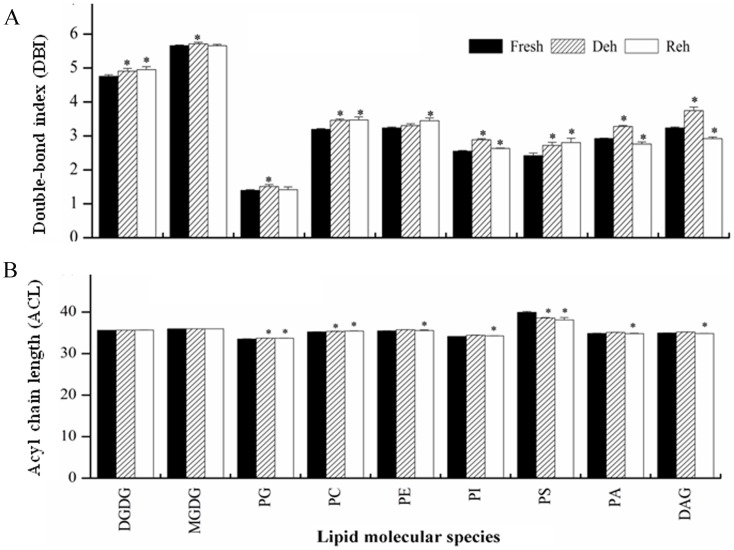
Changes in the (A) double-bond index (DBI) and (B) acyl chain length (ACL) of membrane lipids during dehydration (Deh) and rehydration (Reh) in *P. mileense* leaves. 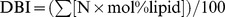
, where N is the total number of double bonds in the two fatty acid chains of each glycerolipid molecule. 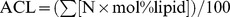, where N is the total number of C atoms in the lipid molecular of each glycerolipid molecule. Bars with asterisks are significantly different (*P* < 0.05). Values are means ± standard deviation (*n*  =  4 or 5).

Membrane fluidity is determined primarily by the level of lipid unsaturation [42] and the acyl chain lengths (ACL) [69] of the lipids. We calculated the double bond index (DBI) and ACL of membrane lipids to determine whether membrane fluidity responds to dehydration and rehydration in *P. mileense* and, if it does, to investigate how this occurs. During dehydration, the DBI of all lipid classes increased significantly (Figure 4A). For example, the DBI of DGDG increased 3.2%, from 4.76 to 4.91, and the DBI of PC increased 8.5%, from 3.19 to 3.46 (Table S2). During rehydration, the DBIs of DGDG, PC, PE, and PS remained high, and the DBIs of PI, PA, and DAG returned to the levels observed in the control (Figure 4A). The ACLs of lipids remained unchanged under most conditions, but some of them increased or decreased slightly during dehydration or rehydration (Figure 4B). These results show that *P. mileense* enhanced membrane fluidity by increasing the level of unsaturation of membrane lipids in order to deal with dehydration. These findings are consistent with observations in other plants, such as *Sporobolus stapfianus* [44], [45] and *Boea hygroscopica* [46].

### Lethal dehydration and rehydration dramatically increased levels of DAG in *P. mileense* although PA levels remained low

**Figure 5:**
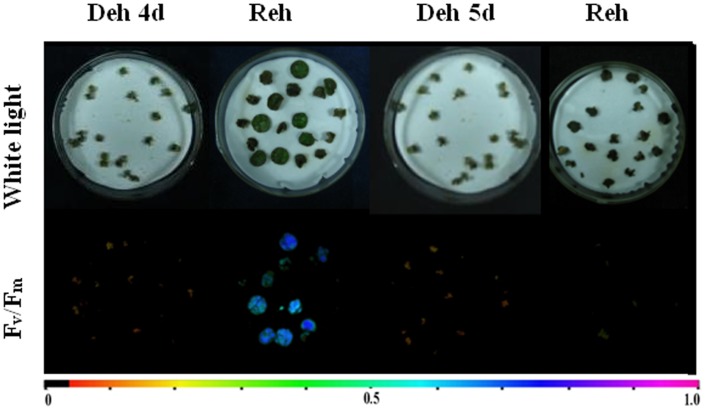
Half-lethal dehydration (Deh 4 d), lethal dehydration (Deh 5 d), and subsequent rehydration (Reh) of *P. mileense* leaves. White coloration (upper picture) or low *F_v_*/*F_m_* values for variable fluorescence (lower picture). The color bar at the bottom indicates *F_v_*/*F_m_* values.

We then investigated the responses of membrane lipids to lethal dehydration in *P. mileense*. We dehydrated leaf discs of *P. mileense* for four days and five days, and then rehydrated them for one day. The four-day dehydration caused more than 70% of the leaf discs to lose their ability to revive, whereas all discs lost their capacity to revive after a five-day dehydration treatment (Figure 5). These findings indicate that persistent dehydration for four days causes lethal damage in the leaf discs of *P. mileense*. We found that, except for PA and DAG, all membrane lipids underwent significant degradation (Table 3). For example, the MGDG content decreased to 6.48 nmol/mg (Table 3), which was only about 10% of the level in fresh discs (Table 2), and PC content decreased to 0.51 nmol/mg (Table 3), which was also only about 10% of that of the fresh discs (Table 2). The PA level remained stable at a low level close to that of discs not subjected to a lethal treatment (Table 2); this differs from findings for the resurrection plant *Craterostigma plantagineum* [37]. In contrast, the DAG level increased dramatically following dehydration, reaching 2.5-fold that of fresh discs (Table 2). These results indicate that membrane deterioration occurred during lethal dehydration, and suggest that lipid degradation occurred through DAG pools and did not involve PA pools. This means that the patterns of lethal dehydration-induced degradation in *P. mileense* were distinct from those in *A. thaliana*.

**Table 3:** Lipid content in each head-group class and total polar lipid during half-lethal (Deh 4 d) or lethal dehydration (Deh 5 d) and rehydration for one day after dehydration for five days (Reh) in *P. mileense* leaves.

Lipid class	Lipid/dry weight (nmol/mg)		
	Deh 4 d	Deh 5 d	Reh
**DGDG**	25.27±1.62^a^	21.10±5.79^a^	12.28±1.99^b^
**MGDG**	15.51±1.62^a^	13.31±2.98^a^	6.48±1.46^b^
**PG**	4.15±0.69^a^	3.4±0.93^a^	2.46±0.32^b^
**PC**	4.86±0.61^a^	4.84±1.38^a^	0.51±0.15^b^
**PE**	0.69±0.10^a^	0.70±0.37^a^	0.04±0.03^b^
**PI**	3.83±0.38^a^	2.70±0.78^b^	0.88±0.23^c^
**PS**	0.04±0.02^a^	0.05±0.03^a^	0.01±0.01^b^
**PA**	0.73±0.20^a^	0.52±0.31^a^	0.52±0.13^a^
**LPG**	0.10±0.02^c^	0.29±0.07^a^	0.19±0.07^b^
**LPC**	0.35±0.04^a^	0.28±0.08^b^	0.04±0.02^c^
**LPE**	0.10±0.02^a^	0.07±0.04^a^	0.01±0.00^b^
**DAG**	31.30±4.08^a^	16.94±5.41^b^	28.15±5.87^a^
**Total polar lipid**	57.18±6.81^a^	47.36±12.32^a^	23.43±4.21^b^

Values in the same row with different letters are significantly different (*P*<0.05). Values are means ± standard deviation (*n* = 4 or 5).

### PLD activity in *P. mileense* is much lower than that in *A. thaliana* under control, dehydration, and rehydration conditions

**Figure 6:**
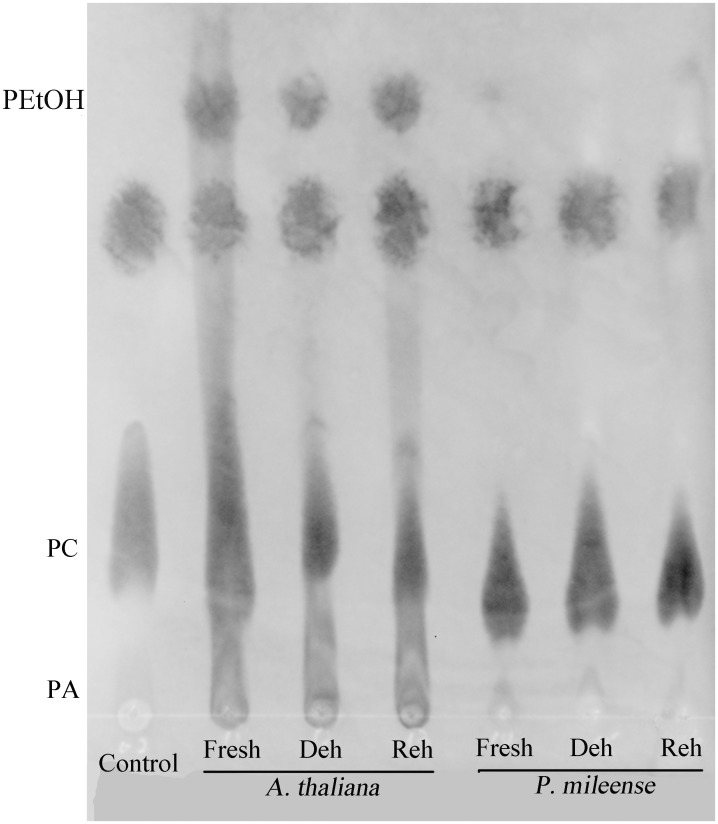
Transphosphatidylation activities of *A. thaliana* and *P. mileense*. Reaction products were separated by thin-layer chromatography and monitored by UV colorimetric analysis.

Given that PLD-mediated hydrolysis makes the greatest contribution to PA formation during water stress [37], [42], [47], [48], we examined PLD activity to explore the reason why *P. mileense* maintains low levels of PA. We used thin-layer chromatography (TLC) to measure the levels of phosphatidylethanol (PEtOH), which is derived from a PLD-specific transphosphatidylation reaction in the presence of PC and ethanol [70]. Proteins isolated from fresh, dehydrated, and rehydrated leaves of *A. thaliana* produced significant amounts of PEtOH (Figure 6). Less PEtOH was derived from the protein extracted from dehydrated leaves than that from protein extracts prepared from fresh and rehydrated leaves of *A. thaliana*. These data indicate that dehydration reduced the substantial level of PLD activity in hydrated *A. thaliana* tissues. In contrast, proteins isolated from fresh, dehydrated, and rehydrated leaves of *P. mileense* produced very much less PEtOH than was produced by comparably treated *A. thaliana* leaves. The results were consistent with the observed changes in PA levels (Table 2), and demonstrated that *P. mileense* was absent in PLD activity.

## Discussion

The mechanisms responsible for the remarkable responses of resurrection plants to desiccation, particularly their changes of membrane lipids, have been reported extensively. However, the diversity of their habitats means that these plants likely adopt different strategies to adapt to desiccation. The novel and distinctive mechanisms of tolerance anticipated for plants indigenous to certain specific areas might provide considerable insight into the mechanisms of adaptation to environments where extreme desiccation occurs frequently. In the present study, we demonstrated that *P. mileense* is a resurrection plant. *P. mileense*, which grows on rocky outcrops with a six-month dry season in southwest China (a region famous for its plant diversity), could revive after desiccation below 10% RWC and showed several physiological and biochemical phenomena typical of resurrection plants. These include progressively inwardly curled leaves, less ion leakage than that observed in desiccation-sensitive plants, more soluble sugar accumulation than that observed in desiccation-sensitive plants, and no proline accumulation during dehydration.

We profiled changes in the composition of membrane lipids of *P. mileense* under non-lethal and lethal desiccation and subsequent rewatering. We also used the desiccation-sensitive plant *A. thaliana* for comparison in terms of both physiological and biochemical analyses. During non-lethal desiccation and subsequent rewatering, *P. mileense* responded by decreasing the abundance of MGDG and increasing the level of lipid unsaturation. Nonetheless, levels of its extraplastidic lipids remained largely unchanged; this response might prevent plasma membrane leakage. In particular, PA and DAG were maintained at low levels similar to those of fresh plants. Upon lethal desiccation, lipid composition decreased substantially owing to dramatic degradation, with large decreases in DGDG, MGDG, PE, PC, PS, PG, and PI and a large increase in DAG; however, the PA content remained low. The level of desiccation that was non-lethal to *P. mileense* was lethal for *A. thaliana*, in which the lipids were massively degraded. The degradation of lipids upon rehydration was more severe than that upon dehydration in *A. thaliana*; all degradation might have occurred through the PA and DAG pools in this species. Interestingly, there was no evidence of PLD activity in *P. mileense*. Our evidence thus indicates that *P. mileense* has two distinguishing features. One is that levels of extraplastidic lipids were stably maintained during non-lethal desiccation. The other is that PA was not involved in the process of lipid degradation, even following lethal membrane damage. These distinctive features might contribute to the capacity of *P. mileense* for resurrection upon rehydration after extreme desiccation.

Tolerance and avoidance are two basic strategies by which plants resist environmental stresses. The model of tolerance in resurrection plants was previously described as a two-step process [42]: destruction during desiccation and recovery from this destruction during rewatering. At cellular levels, the two-step process is like that the membrane lipid composition was damaged and then was reconstituted subsequently. A good example is provided in a recent report on *Craterostigma plantagineum* [37], in which membrane lipids changed during desiccation and returned to the levels observed in controls after rehydration. Changes in PA content were representative, increasing seven-fold and then quickly returning close to normal during dehydration and rehydration, respectively [37]. According to this model, resurrection depends mainly on the capacity of plants to repair the dehydration-induced damage rapidly during rehydration. In contrast, *P. mileense*, the species identified as a resurrection plant in the current study, uses tolerance to resist desiccation by maintaining the composition of the plasma membrane under severe dehydration (Table 2). Such desiccation is lethal to non-resurrection plants (Table 3). Thus, this study has revealed a completely novel model for tolerance in resurrection plants, in which the plasma membrane is maintained through severe dehydration. Damage is not repaired, and if dehydration becomes extreme, the plasma membrane collapses.

This “maintenance or collapse” model of desiccation tolerance is not only of interest to understand how *P. mileense* uses variations in the levels of extraplastidic lipids to deal with desiccation. The current study also raises questions regarding the underlying biochemical mechanisms and whether the effectiveness of the response is linked to the failure of *P. mileense* to increase the PA content, even following massive lipid degradation and membrane deterioration. Phospholipases are major regulators of membrane-lipid metabolism. The significant increases in lyso-phospholipids (Tables 2 and 3) suggest the involvement of phospholipases A and B in the response of *P. mileense* to desiccation. Given that the acyl structures of the accumulated DAG molecules did not match those of the phospholipids degraded in *P. mileense* and *A. thaliana*, DAG was not derived from PLC-mediated phospholipid hydrolysis. Therefore, PLC might not function in the desiccation-induced lipid metabolism, at least not directly. PA pools are a key feature in the lipid degradation pathway. PLD-mediated PA generation is the major pathway of PA formation following water-deficit [37], [42], [47], [71]. As such, a stable low PA content, even after membrane deterioration (Table 2), suggested that *P. mileense* had limited PLD activity, as demonstrated by the transphosphatidylation assay (Figure 6). Another question raised here is that whether maintenance of extraplastidic lipids is the cause or the consequence of desiccation tolerance in *P. mileense*. Our evidence strongly implies that it is a protective mechanism against desiccation. Given that a limit in the size of PA pools can block lipid degradation and thus resist stress-induced damages on the membranes [72], the absence of PLD activity should inhibit lipid degradation during desiccation in *P. mileense*. This might enable *P. mileense* to maintain its extraplastidic lipid composition during desiccation.

In *A. thaliana*, desiccation caused significant lipid changes, but rewatering was associated with extensive lipid degradation (Table 2). In addition, the changes in lipid composition during rewatering were more complicated than those during desiccation (Figure 3B). Given that lethal cellular damage occurred during desiccation, these lines of evidence suggest that lethal damage and the degradation of the most abundant lipids are not synchronous. The reasons for this could be that the consequences of lethal damage developed during rewatering and that severe dehydration not only impacted on the cellular membrane but also reduced enzyme activity, including the activity of lipolytic enzymes. The latter attenuates lipid degradation. Recovery of the activity of lipolytic enzymes, such as PLD, upon rewatering caused substantial lipid hydrolysis in *A. thaliana*.

In summary, we have demonstrated that *P. mileense* is a resurrection plant and that it has a distinct manner of adjusting its membrane lipid composition to tolerate desiccation. *P. mileense* could maintain its extraplastidic lipids until lethal damage occurred. A stable low level of PA, which is associated with limited PLD activity regardless of whether *P. mileense* is subjected to non-lethal or lethal desiccation, accounts for its capacity to maintain the composition of its plasma membranes in order to tolerate severe dehydration. This paper thus presents a novel model of tolerance in resurrection plants in which repair does not occur during rewatering.

## Materials and Methods

### Ethics statement

The habitat of *P. mileense* used in this study is neither a site of conservation of the natural environment nor a private location. Access to this area did not require permission, and the species is not protected by Chinese law when we collected them in January 2008.

### Plant materials

Specimens of the desiccation-tolerant plant *P. mileense* were collected from their natural habitat in Southern Yunnan (24°35′13.8″N, 103°31′59″E, 1960 m alt.), China. There, the plants grow on rocky slopes under trees in a forest. Plants were harvested together with the soil around them. After collection, the *P. mileense* plants were cultivated along with *A. thaliana* seedlings in a chamber at 20–23°C, with a light intensity of 120 µmol m^−2^s^−1^, a 12-h photoperiod, and a relative humidity of 60%. All plants were given adequate water until the beginning of the experiments. Mature and fully expanded leaves were selected for tests. Leaf discs with diameters of 1.5 cm were made from the leaves. Leaf discs of *P. mileense* and *A. thaliana* were each dehydrated for one day by incubation at 15°C in air with a relative humidity of 15%. The dehydrated leaf discs were incubated on wet filter papers in petri dishes for one day at room temperature in the dark to allow them to recover from dehydration.

### Measurement of physiological indices

After dehydration and rehydration treatments, the RWC of leaf discs was determined by weighing them before and after oven drying (105°C for 17 h) and expressed as the percentage water content in water-saturated discs. The RWC of the leaf discs was calculated according to the formula [73]:
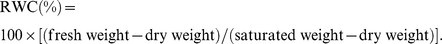



Chlorophyll fluorescence was analyzed using an imaging chlorophyll fluorometer, the MAXI-Imaging Pulse-Amplitude (PAM) instrument (Walz, Germany). After the leaf discs had adapted to darkness for 20 minutes, the maximal quantum yield of photosystem II (PS II) photochemistry was determined on the basis of the initial fluorescence level (*F_0_*) and the maximal fluorescence level (*F_m_*), and was expressed as *F_v_/F_m_* = (*F_m_*−*F_0_*)/*F_m_*.

To determine solute leakage, leaf discs were soaked in 5 ml of double-distilled water and shaken at room temperature for 4 h before aliquots were taken for leachate measurements. Samples were then boiled for 20 min and shaken for an additional hour prior to measuring the maximum conductivity using a DDBJ 350 conductivity meter. The injury index was calculated according to the formula: injury index % = (C/T)×100, where T and C represent the conductivity of the leachate after and before boiling treatment, respectively. To estimate lipid peroxidation (MDA), leaf discs were homogenized in 3 ml of 10% trichloracetic acid. The homogenates were centrifuged at 14,000 *g* for 10 min at 4°C. The MDA content was calculated as described previously [74].

The proline content was determined according to the methods described previously [75] by measuring the quantity of the colored product of proline with ninhydric acid. The absorbance was read at 512 nm. The amount of proline was calculated using a standard curve and expressed as µg/mg dry weight. The solute sugar content was measured by the quantity of the colored reaction product of solute sugar with anthrone. The absorbance was read at 625 nm. The solute sugar content was calculated using a standard curve and expressed as µg/mg dry weight.

### Lipid extraction, ESI-MS/MS, and data processing

The process of lipid extraction, sample analysis, and data processing were performed as described previously, with minor modifications [49], [60]. Briefly, the leaf discs were dropped into 2 ml of isopropanol containing 0.01% butylated hydroxytoluene (BHT) at 75°C. The tissue was extracted three additional times with chloroform:methanol (2∶1) containing 0.01% BHT, with 12 h agitation each time. The remaining plant tissue was dried overnight at 105°C and weighed. Lipid samples were analyzed on a triple quadrupole MS/MS equipped for ESI (Kansas Lipidomics Research Center, http://www.k-state.edu/lipid/lipidomics). The lipids in each class were quantified by comparison with two internal standards of the class. Data processing was performed as described previously [60]. Five replicates of each sampling time were analyzed. The Q test was performed on the total amount of each lipid class with different head group, and outliers were removed [63]. The data were subjected to one-way analysis of variance (ANOVA) with SPSS 16.0. Statistical significance was tested by Fisher's least significant difference (LSD) method. The double-bond index (DBI) was calculated according to the formula: DBI = (Σ[N×mol% lipid])/100, where N is the number of double bonds in each lipid molecule [76]. Acyl chain length (ACL) was calculated using a formula derived from the DBI calculation above: ACL = (Σ [NC×mol% lipid])/100, where NC is the number of acyl carbon atoms in each lipid molecule.

### Protein extraction and PLD activity assay

Protein extraction and assays of PLD activity followed a previous procedure with minor adjustment [70], [77]. Leaf discs were ground into a fine powder under liquid nitrogen. The powder was transferred to a centrifuge tube that contained 0.5 ml of homogenization buffer (50 mM Tris-HCl/pH 7.5, 10 mM KCl, 1 mM EDTA, 2 mM dithiothreitol, and 0.5 mM phenylmethylsulfonyl fluoride). The homogenate was centrifuged at 4800 *g* for 10 min at 4°C. The supernatant was referred to as the soluble total proteins. The total protein content was determined as instructions (Bio-Rad) [78]. The transphosphatidylation reaction mixture contained 100 mM MES (pH 6.5), 25 mM CaCl_2_, 0.5 mM SDS, 1% (v/v) ethanol, enzyme solution containing 30 µg total protein, and 2 mM PC (egg yolk) in a total volume of 150 µl. After adding enzyme solution, the mixture was incubated at 30°C while shaking continuously (100 rpm) for 30 min, and the reaction was stopped by adding 1 ml chloroform: methanol (2∶1) with 0.01% butylatedhydroxytoluene and vortexing the mixture. The chloroform and aqueous phases were separated by adding 100 µl of 2 M KCl. The aqueous phase was transferred into another tube and re-extracted by addition of 0.5 ml of chloroform. The chloroform phases from both tubes were combined and dried. The dried lipid samples were dissolved in 20 µl chloroform, and then spotted onto a TLC plate (silica gel G). The plate was developed with chloroform: methanol: NH_4_OH (6.5∶3.5∶0.2). The spots of PEtOH, PC, and PA were monitored by UV colorimetric analysis after spraying with the color-developing agent primuline [77]. The measurements were repeated twice.

## Supporting Information

Figure S1 **Dehydrated (Deh) and rehydrated (Reh) seedlings of (A)** ***P. mileense*** **and (B)** ***A. thaliana*** **.** White coloration (upper row) or low *F_v_*/*F_m_* values for variable fluorescence (lower row). The color bar at the bottom indicates *F_v_*/*F_m_* values. (C) Relative water content (RWC) values of *P. mileense* and *A. thaliana* following exposure to different periods of dehydration and rehydration.
(TIF)

Figure S2 **Changes in the RWC values of leaf discs during different periods of dehydration of** ***P. mileense*** **and** ***A. thaliana.***
(TIF)

Figure S3 **Changes in the molecular species of membrane lipids following the dehydration (Deh) and rehydration (Reh) of (A)** ***P. mileense*** **and (B)** ***A. thaliana*** **leaves.** Values are means ± standard deviation (*n* = 4 or 5).
(TIF)

Table S1 **Molar percentage of lipids in each head-group class during dehydration (Deh) and rehydration (Reh) in** ***P. mileense*** **and** ***A. thaliana*** **leaves.** The ratios of lipid mol% content of PC/PE and DGDG/MGDG are also shown at the bottom of the table. Values in the same row with different letters are significantly different (*P*<0.05). Values are means ± standard deviation (*n* = 4 or 5).
(DOCX)

Table S2 **Double-bond index (DBI) values of membrane lipids during dehydration (Deh) and rehydration (Reh) in** ***P. mileense*** **and** ***A. thaliana*** **leaves.** DBI = (∑[N×mol% lipid])/100, where N is the total number of double bonds in the two fatty acid chains of each glycerolipid molecule. The percentage relative change in DBI of dehydration RC (F–D) is the value for the difference between the values of Fresh and Deh discs, divided by the value of Fresh discs; that of rehydration RC (D–R) is the value for the significant difference between the values of Deh and Reh discs, divided by the value of Deh discs. Values in the same row with different letters are significantly different (*P*<0.05). Values are means ± standard deviation (*n* = 4 or 5).
(DOCX)
