# Supplementary figures and images for: Maintenance or Collapse: Responses of Extraplastidic Membrane Lipid Composition to Desiccation in the Resurrection Plant Paraisometrum mileense
Source: PLoS One. 2014 Jul 28;9(7):e103430. doi: 10.1371/journal.pone.0103430 (PMC4113352; doi:10.1371/journal.pone.0103430)

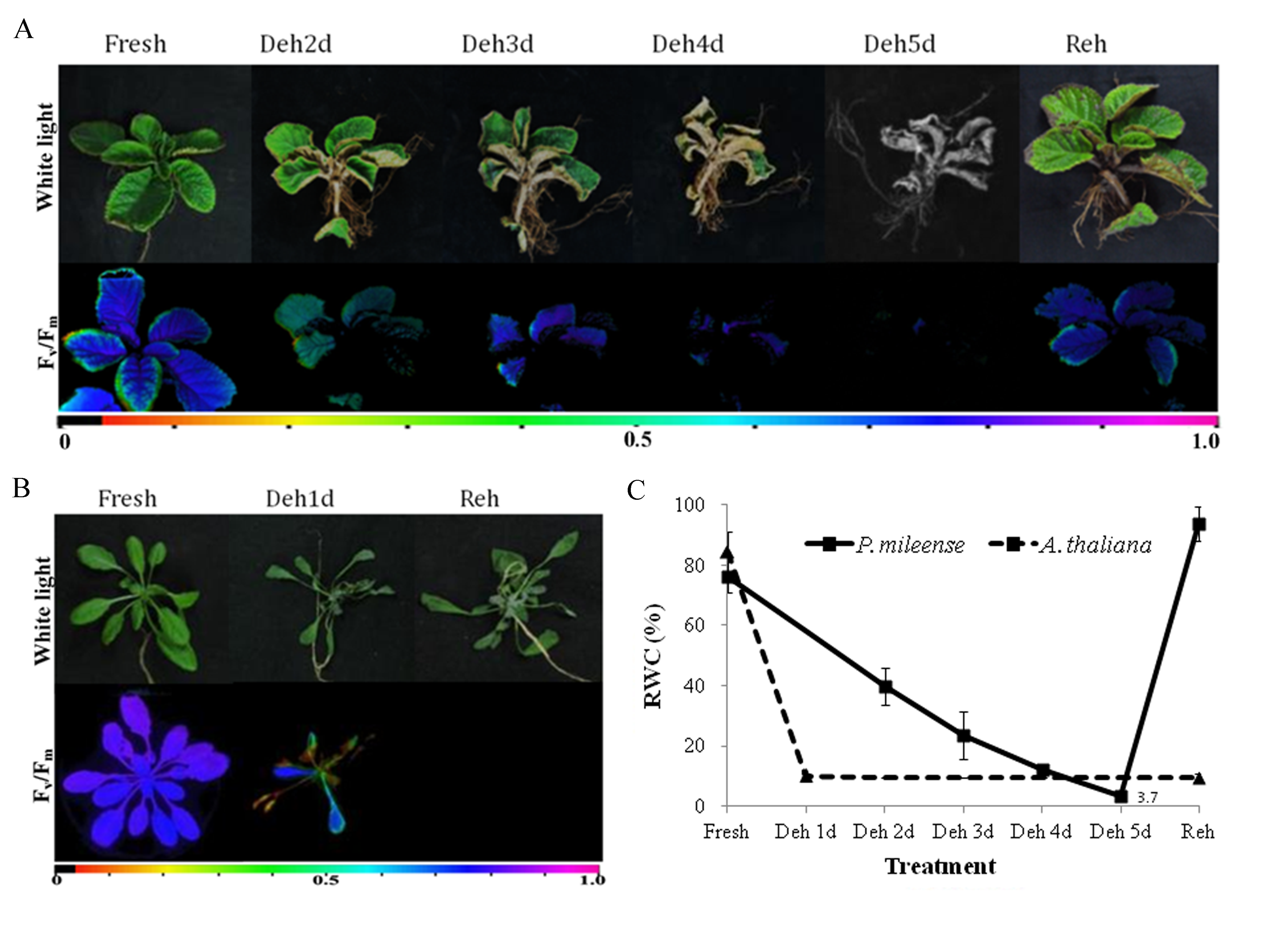

Supplement: Figure S1 — Dehydrated (Deh) and rehydrated (Reh) seedlings of (A) P. mileense and (B) A. thaliana . White coloration (upper row) or low Fv/Fm values for variable fluorescence (lower row). The color bar at the bottom indicates Fv/Fm values. (C) Relative water content (RWC) values of P. mileense and A. thaliana following exposure to different periods of dehydration and rehydration. (TIF) [file pone.0103430.s001.tif]

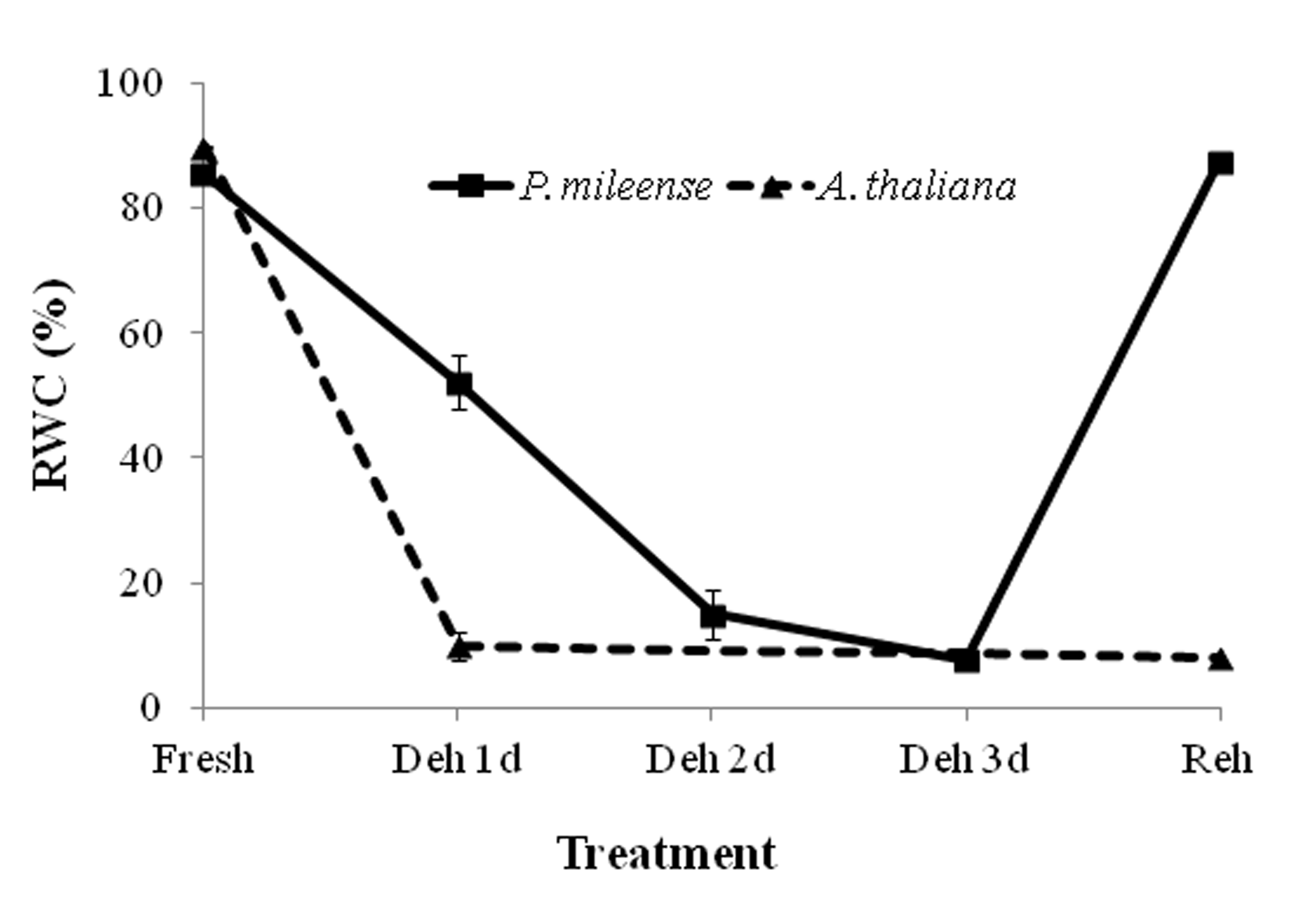

Supplement: Figure S2 — Changes in the RWC values of leaf discs during different periods of dehydration of P. mileense and A. thaliana. (TIF) [file pone.0103430.s002.tif]

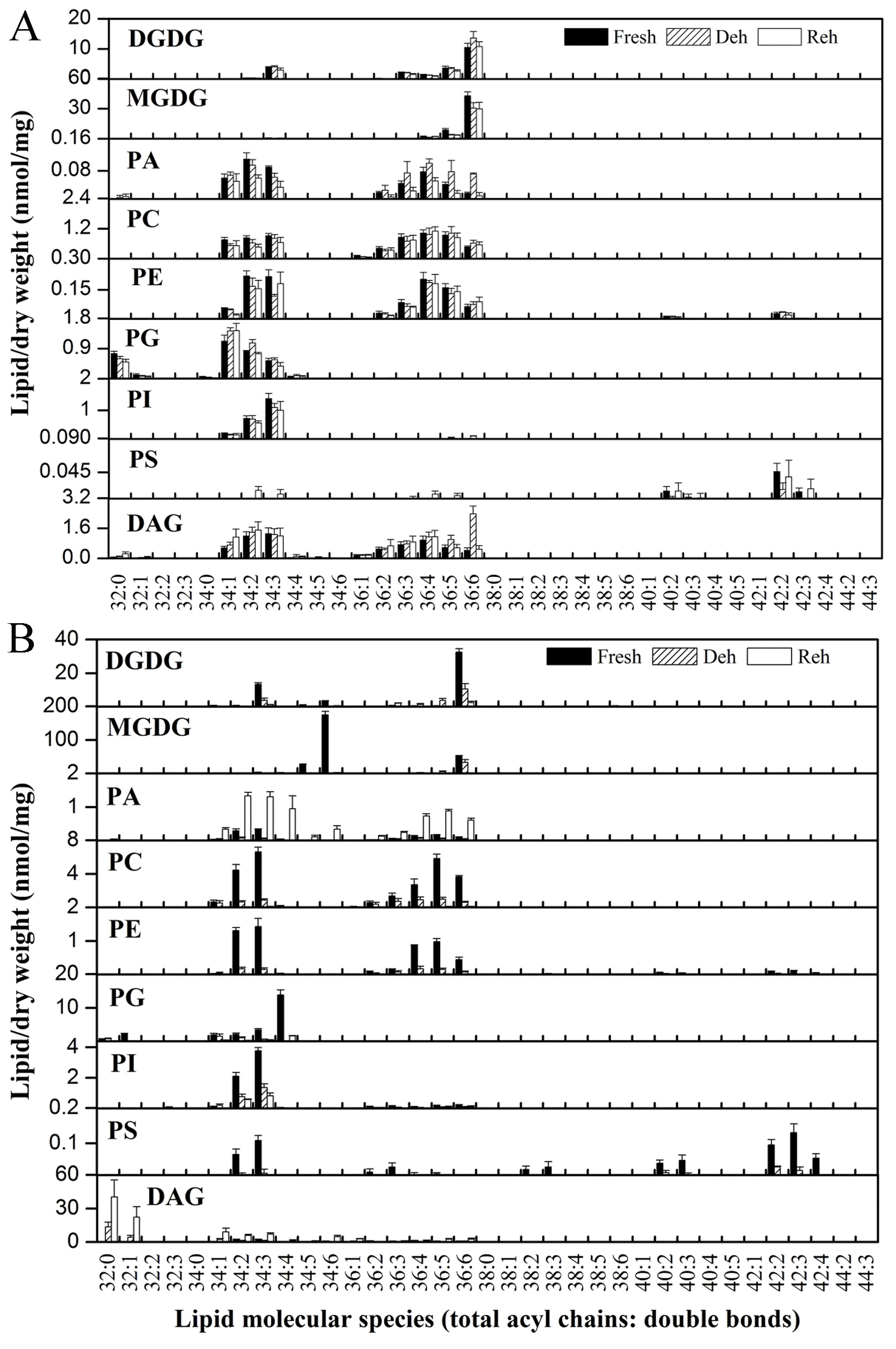

Supplement: Figure S3 — Changes in the molecular species of membrane lipids following the dehydration (Deh) and rehydration (Reh) of (A) P. mileense and (B) A. thaliana leaves. Values are means ± standard deviation (n = 4 or 5). (TIF) [file pone.0103430.s003.tif]
