# Supplementary material for: Maintenance or Collapse: Responses of Extraplastidic Membrane Lipid Composition to Desiccation in the Resurrection Plant Paraisometrum mileense
Source: PLoS One. 2014 Jul 28;9(7):e103430. doi: 10.1371/journal.pone.0103430 (PMC4113352; doi:10.1371/journal.pone.0103430)
Supplement: Table S1 — Molar percentage of lipids in each head-group class during dehydration (Deh) and rehydration (Reh) in P. mileense and A. thaliana leaves. The ratios of lipid mol% content of PC/PE and DGDG/MGDG are also shown at the bottom of the table. Values in the same row with different letters are significantly different (P<0.05). Values are means ± standard deviation (n = 4 or 5). (DOCX) [file pone.0103430.s004.docx]

|  |  |  |  |  |
| --- | --- | --- | --- | --- |
| **Lipid class** | **Species** | **Lipid/total lipid (mol%)** | | |
|  |  |  |  |  |
|  |  |  |  |  |
|  |  | **Fresh** | **Deh** | **Reh** |
|  |  |  |  |  |
|  |  |  |  |  |
| **DGDG** | *A. thaliana* | 13.73 ± 0.46^b^ | 21.06 ± 3.82^a^ | 5.57 ± 3.30^c^ |
|  | *P. mileense* | 22.57 ± 0.38^b^ | 29.26 ± 1.66^a^ | 25.27 ± 1.00^b^ |
|  |  |  |  |  |
| **MGDG** | *A. thaliana* | 66.83 ± 0.49^a^ | 39.65 ± 3.23^b^ | 6.48 ± 3.37^c^ |
|  | *P. mileense* | 55.88 ± 1.28^a^ | 42.82 ± 2.03^c^ | 47.82 ± 3.66^b^ |
|  |  |  |  |  |
| **PG** | *A. thaliana* | 6.04 ± 0.40^a^ | 3.83 ± 0.71^b^ | 2.62 ± 0.57^c^ |
|  | *P. mileense* | 3.47 ± 0.13^b^ | 4.68 ± 0.31^a^ | 4.28 ± 0.43^a^ |
|  |  |  |  |  |
| **PC** | *A. thaliana* | 6.74 ± 0.51^a^ | 5.59 ± 1.19^a^ | 0.33 ± 0.20^b^ |
|  | *P. mileense* | 6.46 ± 0.16^b^ | 6.65 ± 0.96^a^ | 6.93 ± 0.83^a^ |
|  |  |  |  |  |
| **PE** | *A. thaliana* | 1.46 ± 0.13^a^ | 0.90 ± 0.24^b^ | 0.01 ± 0.01^c^ |
|  | *P. mileense* | 1.10 ± 0.21^b^ | 1.07 ± 0.15^b^ | 1.24 ± 0.16^a^ |
|  |  |  |  |  |
| **PI** | *A. thaliana* | 1.77 ± 0.07^b^ | 2.60 ± 0.46^a^ | 1.68 ± 0.51^b^ |
|  | *P. mileense* | 2.68 ± 0.17^a^ | 3.03 ± 0.36^a^ | 2.78 ± 0.38^a^ |
|  |  |  |  |  |
| **PS** | *A. thaliana* | 0.18 ± 0.03^a^ | 0.14 ± 0.05^a^ | 0.00 ± 0.00^b^ |
|  | *P. mileense* | 0.16 ± 0.02^b^ | 0.10 ± 0.02^c^ | 0.27 ± 0.08^a^ |
|  |  |  |  |  |
| **PA** | *A. thaliana* | 0.30 ± 0.04^b^ | 0.42 ± 0.08^b^ | 4.55 ± 1.63^a^ |
|  | *P. mileense* | 0.47 ± 0.07^b^ | 0.64 ± 0.05^a^ | 0.41 ± 0.12^b^ |
|  |  |  |  |  |
| **LPG** | *A. thaliana* | 0.06 ± 0.02^b^ | 0.31 ± 0.10^b^ | 3.08 ± 2.40^a^ |
|  | *P. mileense* | 0.05 ± 0.02^a^ | 0.07 ± 0.02^a^ | 0.04 ± 0.02^a^ |
|  |  |  |  |  |
| **LPC** | *A. thaliana* | 0.02 ± 0.00^c^ | 0.08 ± 0.02^b^ | 0.17 ± 0.02^a^ |
|  | *P. mileense* | 0.04 ± 0.00^b^ | 0.09 ± 0.01^a^ | 0.04 ± 0.01^b^ |
|  |  |  |  |  |
| **LPE** | *A. thaliana* | 0.03 ± 0.00^a^ | 0.03 ± 0.00^a^ | 0.02 ± 0.00^a^ |
|  | *P. mileense* | 0.02 ± 0.01^b^ | 0.07 ± 0.02^a^ | 0.03 ± 0.01^b^ |
|  |  |  |  |  |
| **DAG** | *A. thaliana* | 3.69 ± 1.75^c^ | 25.41 ± 8.05^b^ | 75.10 ± 5.61^a^ |
|  | *P. mileense* | 6.99 ± 1.27^b^ | 11.32 ± 2.33^a^ | 10.90 ± 3.65^a^ |
|  |  |  |  |  |
| **Ratio of lipid mol% content** | | | | |
|  |  |  |  |  |
| **PC/PE** | *A. thaliana* | 4.66 ± 0.55^b^ | 6.01 ± 0.12^b^ | 50.37 ± 8.55^a^ |
|  | *P. mileense* | 5.70 ± 0.56^a^ | 6.24 ± 0.73^a^ | 5.59 ± 0.51^a^ |
|  |  |  |  |  |
| **DGDG/MGDG** | *A. thaliana* | 0.20 ± 0.00^c^ | 0.53 ± 0.08^b^ | 0.83 ± 0.11^a^ |
|  | *P. mileense* | 0.40 ± 0.01^c^ | 0.66 ± 0.01^a^ | 0.53 ± 0.06^b^ |
|  |  |  |  |  |
