# Supplementary material for: Maintenance or Collapse: Responses of Extraplastidic Membrane Lipid Composition to Desiccation in the Resurrection Plant Paraisometrum mileense
Source: PLoS One. 2014 Jul 28;9(7):e103430. doi: 10.1371/journal.pone.0103430 (PMC4113352; doi:10.1371/journal.pone.0103430)
Supplement: Table S2 — Double-bond index (DBI) values of membrane lipids during dehydration (Deh) and rehydration (Reh) in P. mileense and A. thaliana leaves. DBI = (∑[N×mol% lipid])/100, where N is the total number of double bonds in the two fatty acid chains of each glycerolipid molecule. The percentage relative change in DBI of dehydration RC (F–D) is the value for the difference between the values of Fresh and Deh discs, divided by the value of Fresh discs; that of rehydration RC (D–R) is the value for the significant difference between the values of Deh and Reh discs, divided by the value of Deh discs. Values in the same row with different letters are significantly different (P<0.05). Values are means ± standard deviation (n = 4 or 5). (DOCX) [file pone.0103430.s005.docx]

|  |  |  |  |  |  |  | |
| --- | --- | --- | --- | --- | --- | --- | --- |
| **Lipid class** | **Species** | **Double bonding index (DBI)** | | | | | |
|  |  |  |  |  |  | |  |
|  |  |  |  |  |  | |  |
|  |  | **Fresh** | **Dehydrated** | **Rehydrated** | **RC(F-D)(%)** | | **RC(D-R)(%)** |
|  |  |  |  |  |  | |  |
|  |  |  |  |  |  | |  |
| **DGDG** | *A. thaliana* | 5.06 ± 0.05^a^ | 4.77 ± 0.09^b^ | 4.99 ± 0.12^a^ | –5.7 | | 4.6 |
|  | *P. mileense* | 4.76 ± 0.05^b^ | 4.91 ± 0.08^a^ | 4.95 ± 0.09^a^ | 3.2 | | 0.8 |
|  |  |  |  |  |  | |  |
| **MGDG** | *A. thaliana* | 5.82 ± 0.02^a^ | 5.69 ± 0.05b^b^  ^b^ 2b | 5.74 ± 0.08^b^ | –2.2 | | 0.9 |
|  | *P. mileense* | 5.66 ± 0.02^b^ | 5.71 ± 0.05^a^ | 5.66 ± 0.04^b^ | 0.9 | | –0.9 |
|  |  |  |  |  |  | |  |
| **PG** | *A. thaliana* | 3.09 ± 0.04^a^ | 1.41 ± 0.06^b^ | 3.14 ± 0.11^a^ | –54.4 | | 122.7 |
|  | *P. mileense* | 1.39 ± 0.02^b^ | 1.51 ± 0.06^a^ | 1.42 ± 0.08^b^ | 8.6 | | –6 |
|  |  |  |  |  |  | |  |
| **PC** | *A. thaliana* | 3.75 ± 0.09^a^ | 3.43 ± 0.11^b^ | 3.82 ± 0.18^a^ | –8.5 | | 11.4 |
|  | *P. mileense* | 3.19 ± 0.02^b^ | 3.46 ± 0.04^a^ | 3.47 ± 0.09^a^ | 8.5 | | 0.3 |
|  |  |  |  |  |  | |  |
| **PE** | *A. thaliana* | 3.46 ± 0.04^a^ | 3.4 ± 0.09^a^ | 2.9 ± 0.68^b^ | –1.7 | | –14.7 |
|  | *P. mileense* | 3.24 ± 0.02^b^ | 3.3 ± 0.05^b^ | 3.45 ± 0.08^a^ | 1.9 | | 4.5 |
|  |  |  |  |  |  | |  |
| **PI** | *A. thaliana* | 2.81 ± 0.04^b^ | 2.75 ± 0.01^b^ | 2.9 ± 0.08^a^ | –2.1 | | 5.5 |
|  | *P. mileense* | 2.55 ± 0.02^c^ | 2.89 ± 0.03^a^ | 2.63 ± 0.01^b^ | 13.3 | | –9 |
|  |  |  |  |  |  | |  |
| **PS** | *A. thaliana* | 2.83 ± 0.01^ab^ | 2.67 ± 0.03^b^ | 3.41 ± 0.75^a^ | –5.7 | | 27.7 |
|  | *P. mileense* | 2.42 ± 0.08^b^ | 2.72 ± 0.09^a^ | 2.81 ± 0.13^a^ | 12.4 | | 3.3 |
|  |  |  |  |  |  | |  |
| **PA** | *A. thaliana* | 3.36 ± 0.07^b^ | 3.27 ± 0.09^b^ | 3.65 ± 0.03^a^ | –2.7 | | 11.6 |
|  | *P. mileense* | 2.92 ± 0.01^b^ | 3.28 ± 0.03^a^ | 2.76 ± 0.06^c^ | 12.3 | | –15.9 |
|  |  |  |  |  |  | |  |
| **LPG** | *A. thaliana* | 1.81 ± 0.1^a^ | 0.91 ± 0.05^c^ | 1.15 ± 0.09^b^ | –49.7 | | 26.4 |
|  | *P. mileense* | 0.7 ± 0.2^b^ | 1.16 ± 0.21^a^ | 1.27 ± 0.23^a^ | 65.7 | | 9.5 |
|  |  |  |  |  |  | |  |
| **LPC** | *A. thaliana* | 1.88 ± 0.1^a^ | 1.51 ± 0.02^b^ | 1.18 ± 0.21^c^ | –19.7 | | –21.9 |
|  | *P. mileense* | 1.39 ± 0.22^a^ | 1.46 ± 0.12^a^ | 1.65 ± 0.22^a^ | 5 | | 13 |
|  |  |  |  |  |  | |  |
| **LPE** | *A. thaliana* | 1.33 ± 0.16^a^ | 1.42 ± 0.3^a^ | 0.38 ± 0.1^b^ | 6.8 | | –73.2 |
|  | *P. mileense* | 1.16 ± 0.3^a^ | 0.95 ± 0.09^a^ | 0.88 ± 0.32^a^ | –18.1 | | –7.4 |
|  |  |  |  |  |  | |  |
| **DAG** | *A. thaliana* | 3.22 ± 0.26^a^ | 1.06 ± 0.17^c^ | 1.86 ± 0.84^b^ | –67.1 | | 75.5 |
|  | *P. mileense* | 3.24 ± 0.03^b^ | 3.75 ± 0.11^a^ | 2.92 ± 0.05^c^ | 15.7 | | –22.1 |
|  |  |  |  |  |  | |  |
| **Total polar lipid** | *A. thaliana* | 5.29 ± 0.04^a^ | 4.87 ± 0.04^a^ | 4.1 ± 0.58^b^ | –7.9 | | –15.8 |
|  | *P. mileense* | 4.97 ± 0.01^a^ | 4.89 ± 0.08^a^ | 4.93 ± 0.07^a^ | –1.6 | | 0.8 |
|  |  |  |  |  |  | |  |
|  |  |  |  |  |  | |  |
